# STK3 Suppresses Ovarian Cancer Progression by Activating NF-*κ*B Signaling to Recruit CD8^+^ T-Cells

**DOI:** 10.1155/2020/7263602

**Published:** 2020-09-29

**Authors:** Xiangyu Wang, Fengmian Wang, Zhi-Gang Zhang, Xiao-Mei Yang, Rong Zhang

**Affiliations:** ^1^The Third School of Clinical Medicine, Southern Medical University, Guangzhou 510500, China; ^2^Department of Obstetrics and Gynecology, Fengxian Central Hospital Affiliated to the Southern Medical University, Shanghai 201499, China; ^3^State Key Laboratory of Oncogenes and Related Genes, Shanghai Cancer Institute, Ren Ji Hospital, School of Medicine, Shanghai Jiao Tong University, Shanghai 201109, China

## Abstract

Serine/threonine protein kinase-3 (STK3) is a critical molecule of the Hippo pathway but little is known about its biological functions in the ovarian cancer development. We demonstrated the roles of STK3 in ovarian cancer. Existing databases were used to study the expression profile of STK3. STK3 was significantly downregulated in OC patients, and the low STK3 expression was correlated with a poor prognosis. In vitro cell proliferation, apoptosis, and migration assays, and in vivo subcutaneous xenograft tumor models were used to determine the roles of STK3. The overexpression of STK3 significantly inhibited cell proliferation, apoptosis, and migration of ovarian cancer cells in vitro and tumor growth in vivo. Bisulfite sequencing PCR analysis was performed to validate the methylation of STK3 in ovarian cancer. RNA sequencing and gene set enrichment analysis (GSEA) were used to compare the transcriptome changes in the STK3 overexpression ovarian cancer and control cells. The signaling pathway was analyzed by western blotting. STK3 promoted the migration of CD8^+^ T-cells by activating nuclear transcription factor *κ*B (NF-*κ*B) signaling. STK3 is a potential predictor of OC. It plays an important role in suppressing tumor growth of ovarian cancer and in chemotaxis of CD8^+^ T-cells.

## 1. Introduction

Ovarian cancer (OC) is the most frequent cause of death among gynecologic malignancies [1]. Due to its asymptomatic development, the disease is frequently diagnosed at advanced and incurable stages [2]. Although there is a high response rate to surgery and chemotherapy, the majority of patients subsequently relapse [3]. Therefore, it is important to identify effective molecular targets influencing tumor progression.

STK3 is a key molecule in the Hippo pathway, which controls cell development, proliferation, apoptosis, and various stress responses [4]. STK3 plays a role in inhibiting the progression of gastric cancer, hepatocellular carcinoma, and breast cancer by activating Hippo signaling [5–7]. STK3 also regulates the immune system in infections [8]. However, it is unclear whether STK3 can exert cancer-suppressing effects by regulating the functions of the immune system.

CD8+ T-cells play an important role in inhibiting the development of ovarian cancer [9]. The chemotaxis of CD8+ T-cells is regulated by a variety of chemokines, of which CXCL16 and CX3CL1 are the most important on CD8+ T-cells [10, 11]. The low expression of CXCL16 in breast cancer, osteosarcoma, renal clear cell carcinoma, and melanoma is associated with a poor prognosis [12–14]. The CX3CL1 expression is increased in the renal clear cell carcinoma tissues of patients receiving bevacizumab, accompanied by infiltration of CD8+ T-cells [15]. NF-*κ*B can affect the infiltration of CD8+ T-cells in pancreatic ductal adenocarcinoma cells by regulating the generation of CXCL16 and CX3CL1 and thereby promoting the apoptosis of pancreatic ductal adenocarcinoma cells [16]. However, it is unclear whether STK3 can activate NF-*κ*B signaling and suppress ovarian cancer growth.

In this study, we demonstrated that STK3 was downregulated in ovarian cancer via epigenetic methylation of its promoter DNA. STK3 inhibited proliferation and metastasis of ovarian cancer cells. The overexpression of STK3 in ovarian cancer cells induced cell cycle arrest at G2/M and a higher rate of cell apoptosis. We also found that STK3 promoted the recruitment of CD8+ T-cells via an increase in chemokine CXCL16 and CX3CL1 production in human ovarian cancer cells. This was possibly mediated by the regulation of the NF-*κ*B signaling pathway. Our results revealed a new molecular mechanism for serous ovarian cancer malignancy mediated by STK3 through regulating NF-*κ*B signaling and CD8+ T-cell recruitment.

## 2. Materials and Methods

### 2.1. Data Mining

The mRNA expression profiles for normal ovarian surface epithelium and ovarian cancer were downloaded from UCSCXena (https://xena.ucsc.edu) and GEO (https://www.ncbi.nlm.nih.gov/geo/). The survival data of patients were downloaded from TCGA (https://www.cancer.gov/about-nci/organization/ccg/research/structural-genomics/tcga) and GSE9899.

### 2.2. Cell Culture and Clinical Samples

IOSE80, OVCAR3, OVCAR8, CAOV3, ES-2, and HEK293T cell lines were all purchased from Cell Bank of the Chinese Academy of Sciences (Shanghai, China). All of the cells were cultured following the instructions of the American Type Culture Collection (ATCC, Manassas, VA, USA).

### 2.3. Reagents

The antibodies used in this study were against STK3 (ab52641, Abcam, Cambridge, MA, USA), Phospho-I*κ*B*α* (#2859, Cell Signaling Technology), I*κ*B*α* (#4812, Cell Signaling Technology), Phospho-NF-*κ*B p65 (#3033, Cell Signaling Technology), *β*-actin (GB11001, Servicebio, Wuhan, China), and NF-*κ*B p65 (#8242, Cell Signaling Technology) for western blotting. Secondary antibodies were purchased from Jackson ImmunoResearch (West Grove, PA, USA). Chemicals and biochemical used were DAC (A3656, Sigma-Aldrich, St. Louis, MO, USA) and TSA (S1045, Selleck, Shanghai, China). CXCL16 and CX3CL1 concentrations were measured in the medium of ovarian cancer cells using enzyme-linked immunosorbent assay (ELISA) kits (R&D Systems, Abingdon, UK) according to manufacturer's instructions.

### 2.4. Quantitative Real-Time PCR

Total RNA was extracted from cells in each group using Trizol kits, and qualified total RNA samples were subjected to reverse transcription. Total RNA was taken to synthesize complementary deoxyribonucleic acid (cDNA) using the PrimeScript™RT reagent Kit (RR047A, TAKARA, Japan). The specific reaction conditions were 37°C for 30 min and 85°C for 30 s. Quantitative analysis was carried out using the ABI 7500 fluorescence PCR amplification instrument (Applied Biosystems; Thermo Fisher Scientific, Inc.). *RPS18* was used as an endogenous control, and Ct values were processed using the 2^−*ΔΔ*Ct^ method. The sequences of primers used in this experiment were shown in Table 1.

### 2.5. Western Blot Analysis

For western blot analysis, total protein samples were extracted from cells using RIPA lysis buffer with a protease inhibitor (Beyotime, Jiangsu, China). An equal amount of protein (60 *μ*g/lane) was loaded on 10% or 8% SDS-polyacrylamide gels and then transferred to a pure nitrocellulose blotting membrane (Pall Life Science, AZ, USA). Next, the membrane was blocked in 5% nonfat milk for 1 h and then probed with primary antibodies against STK3, *β*-actin, Phospho-I*κ*B*α*, I*κ*B*α*, Phospho p65, and p65 overnight at 4°C. Then, the membranes were incubated with anti-rabbit secondary antibodies. Finally, immunoreactivity was detected using the Odyssey Infrared Imaging System (Gene Company Limited, Hong Kong, China).

### 2.6. DAC and TSA Treatment

Cells were treated with 5 or 10 *μ*M of 5-aza-2′-deoxycytidine (DAC, Sigma-Aldrich, St. Louis, MO, USA) or 300 nM trichostatin A (TSA, Selleckchem, TX, USA) for 3 d, and the drug media were replaced every 24 h. Control cells were incubated with the same volume of DMSO. In the combined treatment group, cells were cultured in the presence of 5 *μ*M of DAC for 2 d and were then treated for an additional 24 h with 300 nM of TSA.

### 2.7. Plasmid Construction and Transfection

The human STK3 was subcloned into the pcDNATM3.1 vector. STK3 and the mock vector were packaged into the virus, and titers were determined. Target cells were infected with 1 × 10^8^ lentivirus-transducing units and 6 *μ*g/ml polybrene (TR-1003, Sigma-Aldrich, Germany). After 72 h, the infected cells were screened in the presence of 3 *μ*g/ml puromycin. The qPCR and western blot also verified the overexpression efficacy of STK3.

### 2.8. Cell Viability Assays

Cell viability was determined using a cell counting kit-8 (CK04, Dojindo, Japan). Briefly, water-soluble formazan dye was added to the culture medium and incubated for 2 h in 5% CO_2_. The developed color was measured at 450 nm using an ELISA plate reader.

### 2.9. Colony Formation Assay

The 1 × 10^3^ cells/mL single-cell suspension was made in RPMI1640 or DMEM. The colonies were stained with 0.04% crystal violet and 2% ethanol in PBS and then incubated at 37°C for 21 days. The stained colonies were photographed under a light microscope (Olympus).

### 2.10. Transwell Migration and Invasion

Cells were seeded in the upper chamber of transwell plates (Corning, NY, NY USA) with serum-free medium. 10% FBS medium was added to the lower chamber of the transwell. Next, cells were transfected as described above for 48 h. For the invasion experiments, the upper chamber was covered with culture medium and matrigel (BD Biosciences, San Jose, CA, USA) mixture. Finally, cells on the top of the chamber were removed with cotton swabs, while cells that went through the membrane were stained with 0.5% crystal violet and counted under a microscope at 100x magnification.

### 2.11. Flow Cytometry Test of Cell Cycle

Ten ml of cell suspension (1 − 5 × 10^6^ cells/mL) was spun at 500–1000 rpm for 5 min. The cell pellet was washed with PBS once and fixed in 3.70% ice ethanol at 4°C for 2 h. After washing with PBS twice, the cells were suspended in PBS containing 50 mg/L RNase A and 25 *μ*g/mL PI and incubated in darkness at 25°C for 15 min. The cells at different phases were measured using flow cytometry with an excitation wave length of 488 nm and an emission wave length greater than 560 nm to detect PI signal.

### 2.12. Flow Cytometry Test of Apoptosis

Ten ml of cell suspension (1–5 × 10^6^ cells/mL) was spun at 500–1000 rpm for 5 min. The cell pellet was washed with PBS once and fixed in 70% ice ethanol at 4°C for 2 h. After the fixation solution was removed, the cells were suspended in 3 ml PBS for 5 min. Cells were transfected with lentivirus for 48 h and serum-free starved for 24 h. This was filtered once with a 400 gauge filter and centrifuged at 1000 rpm for 5 min, and then the cell pellet was stained with 50 mg/mL propidium iodide and Annexin V–fluorescein isothiocyanate (BD Pharmingen, Franklin Lakes, NJ, USA) following manufacturer's instructions. Both the living and dead cells were measured using flow cytometry with an exciting wave length of 488 nm and an emission wave length greater than 630 nm.

### 2.13. Xenograft Transplantation

Female nude (BALB/c) mice (4 weeks old) were purchased from the East China Normal University. Mice were randomly divided into two groups. OS cells stably expressing vector or lenti-STK3 were propagated, and 1 × 10^7^ cells were inoculated subcutaneously into the right side of the posterior flank of the mice. Tumor growth was examined at the indicated time points, and tumor volumes were measured. After 4 weeks, the mice were killed, weighed, excised, fixed, and embedded in paraffin and sectioned for the histological examination of the PCNA and TUNEL expression.

### 2.14. Statistical Analysis

Data are presented as means ± SD. Statistical analyses were done using SPSS V.16.0 for Windows (IBM) or GraphPad Prism (GraphPad Software, San Diego, CA, USA). Cumulative survival time was calculated by the Kaplan-Meier method and analyzed by the log-rank test. Correlation of the STK3 expression with clinical parameters in patients with ovarian cancer was evaluated by *χ*^2^ test. Correlation was determined using the Spearman's test. Student's *t*-test was used for paired comparisons between groups. *P* values <0.05 were considered to be statistically significant.

## 3. Results

### 3.1. STK3 Was Downregulated in Ovarian Cancer and Correlated with Prognosis

**Figure 1:**
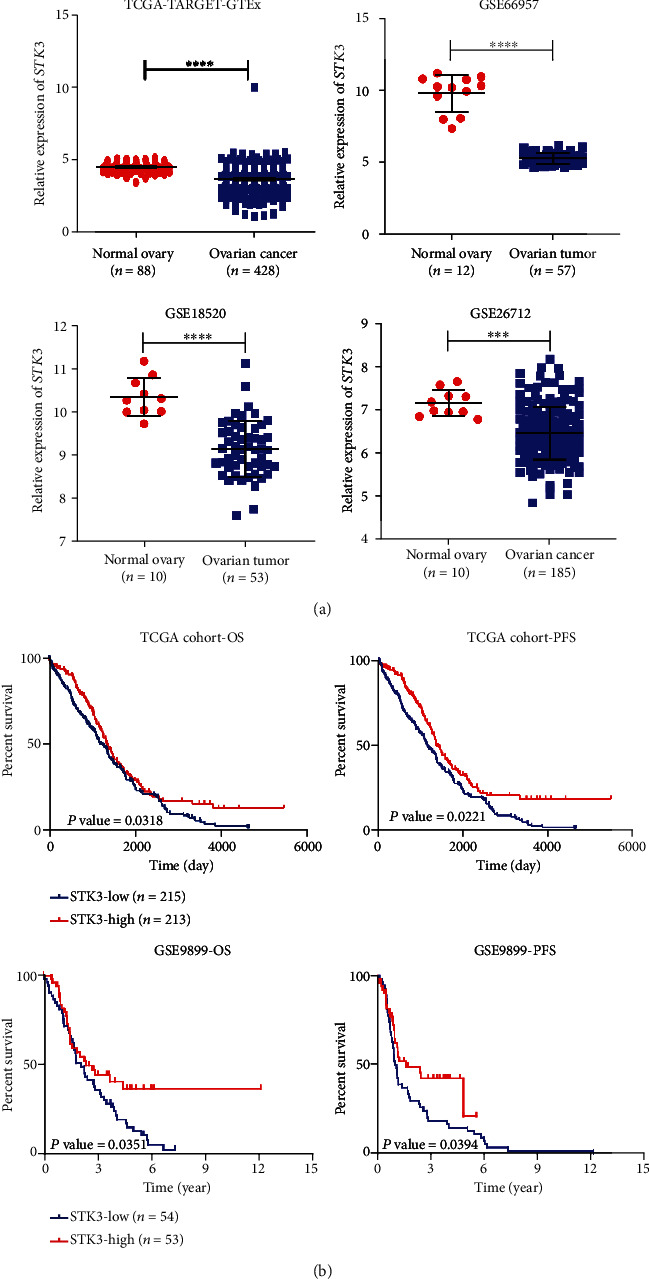
Database analysis showed that STK3 was downregulated in ovarian cancer tissues, and the overexpression of STK3 was closely related to better prognosis. (a) Analysis of the mRNA level expression of STK3 in the TCGA database and GEO database (two-tailed Student's *t*-test, ^∗∗∗^*P* < 0.001; ^∗∗∗∗^*P* < 0.0001). (b) Analysis of the overall survival period and progression-free survival period according to the STK3 expression in the TCGA database and GEO database (log-rank test).

To investigate the level of STK3 in ovarian cancers, we first compared the expression of STK3 in ovarian cancer and matching normal ovarian surface epithelial tissue samples via GTEx and TCGA. Notably, STK3 was significantly downregulated in ovarian cancer based on the analysis of TCGA and GEO datasets (*P* value <0.05) (Figure 1(a)). We also investigated whether the STK3 expression was correlated with prognosis in ovarian cancer patients. In TCGA cohorts, which included 428 samples of ovarian cancer, the low STK3 expression was associated with poorer overall survival and progression-free survival prognosis (*P* value <0.05) (Figure 1(b)). The poor prognosis in ovarian cancer also correlated with the lower STK3 expression in GSE9899 (*P* value <0.05) (Figure 1(b)). These results suggest that the STK3 expression influences the progression of ovarian cancer.

### 3.2. The Overexpression of STK3 Inhibited the Proliferation, Migration, and Invasion of Ovarian Cancer Cells

**Figure 2:**
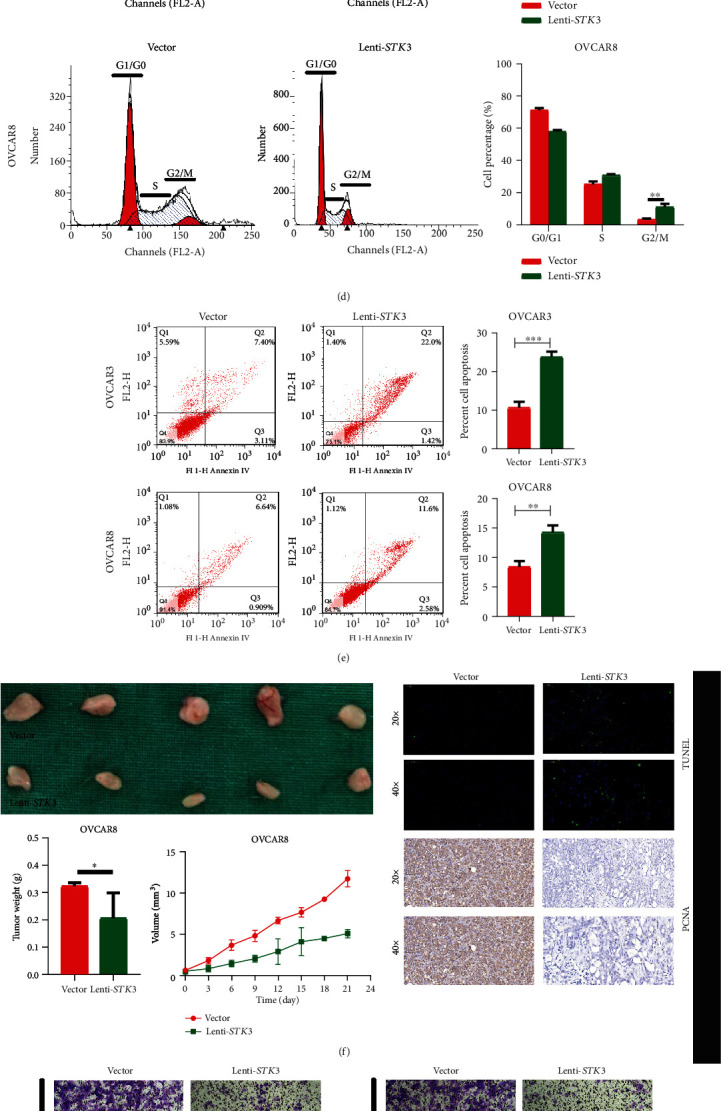
Overexpressed STK3 can inhibit the invasion and proliferation of ovarian cancer cells in vitro and in vivo. (a) The STK3 expression in five different cell lines measured by qRT-PCR and Western blotting. (b) The STK3 overexpression was confirmed by Western blotting and qRT-PCR in OVCAR3 and OVCAR8 cell lines. (c) Effect of the STK3 overexpression on the proliferation and colony formation of OVCAR3 and OVCAR8 cells in vitro (two-tailed Student's *t*-test, ^∗^*P* < 0.05; ^∗∗∗^*P* < 0.001). (d) The overexpression of STK3 arrested ovarian cancer cells at G2/M. The cell cycle distribution of OVCAR3 and OVCAR8 cells detected by flow cytometry (two- tailed Student's *t*-test, ^∗^*P* < 0.05). (e) The overexpression of STK3 promotes ovarian cancer cell apoptosis, as detected by cell apoptosis assays (two- tailed Student's *t*-test, ^∗∗^*P* < 0.01; ^∗∗∗^*P* < 0.001). (f) Morphologic characteristics of tumors from mice inoculated with vector and lenti-STK3/OVCAR8 cells. Tumor weights and volumes of the vector and lenti-STK3 groups are shown (two- tailed Student's *t*-test, ^∗∗^*P* < 0.01). The overexpression of STK3 can inhibit the invasion and migration of ovarian cancer cells. (g) Effect of the STK3 overexpression on invasion and migration of OVCAR3 and OVCAR8 cells by cell migration and invasion assays (two- tailed Student's *t*-test, ^∗∗∗^*P* < 0.001; ^∗∗∗∗^*P* < 0.0001). Scale bar: 100 *μ*m. (h) Effect of the STK3 overexpression on the migration of OVCAR3 and OVCAR8 cells detected by wound healing assays (two- tailed Student's *t*-test, ^∗∗∗^*P* < 0.001; ^∗∗∗∗^*P* < 0.0001). Scale bar: 100 *μ*m.

To study the expression pattern of STK3 in ovarian cancer, we compared the level of STK3 in a normal human ovarian surface epithelium cell line (IOSE80) with levels in ovarian cancer cell lines (CAOV3, OVCAR3, OVCAR8, and ES-2). The STK3 expression levels detected by western blot were lower in most tested ovarian cancer cells compared with IOSE80 (Figure 2(a)).

To further explore the role of STK3 in ovarian cancer progression in vitro, we established stable STK3-overexpressing cells by lentivirus-mediated transfection. Quantitative real-time polymerase chain reaction (qPCR) and western blotting confirmed the efficiency of the STK3 overexpression in OVCAR3 and OVCAR8 cells (Figure 2(b)). We determined the effect of STK3 on the proliferation of ovarian cancer cells through CCK-8 and colony formation assays. The results showed that, compared with the control group, the proliferation and clone formation ability of ovarian cancer cells OVCAR3 and OVCAR8 were significantly inhibited by overexpressing STK3 (*P* value <0.05) (Figure 2(c)).

We used flow cytometry to detect the effect of STK3 on the ovarian cancer cell cycle distribution. Cell cycle analysis showed that the proportion of OVCAR3 and OVCAR8 cells in the G2/M phase significantly increased after exposure to overexpressing STK3 (*P* value <0.05) (Figure 2(d)).

We determined the effect of STK3 on ovarian cancer cells apoptosis by annexin V/PI staining and flow cytometry analysis in vitro. The results showed that the apoptosis rate of the lenti-STK3 group in OVCAR3 cell line was significantly higher than that of the control group (17.72 ± 0.3358% versus 9.887 ± 0.1203%, *P* value <0.05). Similarly, the apoptosis rate of the lenti-STK3 group in OVCAR8 cell line was also significantly higher than that in control group (*P* value <0.05) (Figure 2(e)).

Finally, lentivector or lenti-STK3 OVCAR8 cells were subcutaneously injected into nude mice to assess in vivo growth of tumors. The results showed that the tumor volume and tumor weight of the lentivector group were larger than those of the lenti-STK3 group. These results indicate that the overexpression of STK3 significantly inhibited the growth of ovarian cancer (*P* value <0.05) (Figure 2(f)).

To determine the effect of STK3 on the migration and invasion in ovarian cancer cells, we first examined the migration of OVCAR3 and OVCAR8 cells via transwell invasion and migration assays after overexpressing STK3. The results showed that, compared with the control group, the invasive and migratory abilities of ovarian cancer cells were significantly decreased after exposure to overexpressed STK3 (*P* value <0.05) (Figure 2(g)).

We also used a wound healing assay to determine the effect of STK3 on the migration of serous ovarian cancer cells OVCAR3 and OVCAR8. The results showed that the migration ability of serous ovarian cancer cells was significantly decreased in the STK3 overexpression group compared with the control group (*P* value <0.05) (Figure 2(h)). Thus, the overexpression of STK3 inhibited the invasion and migration of ovarian cancer cells.

### 3.3. STK3 Is Downregulated in Ovarian Cancer via Epigenetic Promoter DNA Methylation

**Figure 3:**
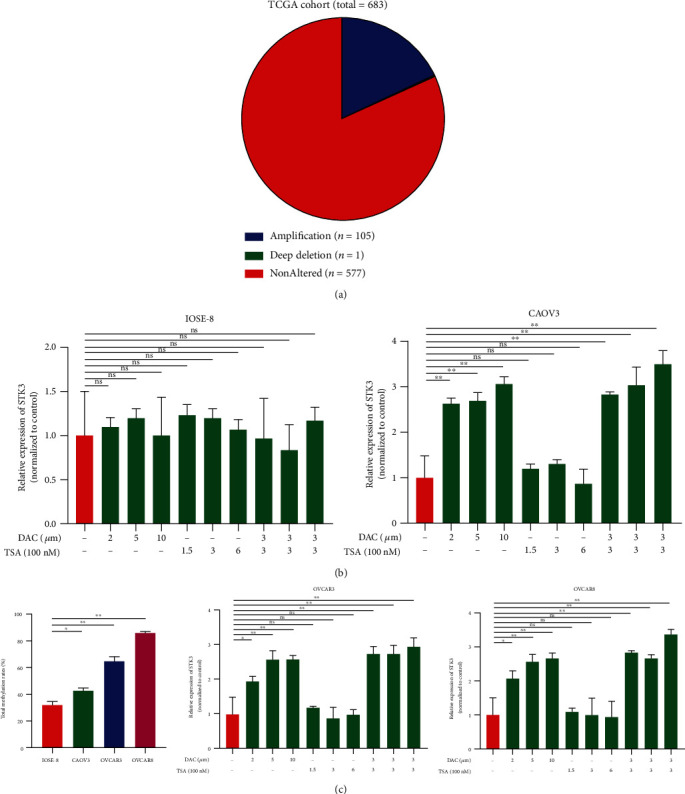
The STK3 expression in ovarian cancer cell lines. (a) Statistics of STK3-mutated samples derived from 683 patients in cBioportal. (b) The mRNA expression of STK3 was evaluated by real-time qPCR in cell lines (IOSE80, CAOV3, OVCAR3, and OVCAR8) treated with DMSO, DAC, TSA, or DAC plus TSA (two-tailed Student's *t*-test, ^∗∗^*P* < 0.01). (c) Total methylation rates of STK3 promoter in the human osteoblast cell line IOSE80 and three ovarian cancer cell lines (CAOV3, OVCAR3, and OVCAR8) are shown. (two-tailed Student's *t*-test, ^∗^*P* < 0.05, ^∗∗^*P* < 0.01).

Genetic or epigenetic mechanisms (or both) might cause dysregulation of the STK3 expression in ovarian cancer. STK3 is located on chromosome 8q22.2, and there is no deletion in this region in ovarian cancer (Figure 3(a)). We explored the epigenetic regulation of the STK3 expression in ovarian cancer. Using 5-aza-20-deoxycytidine (DAC), a specific methyltransferase inhibitor, and Trichostatin A (TSA), a histone deacetylase inhibitor, we found that the mRNA expression level of STK3 in ovarian cancer cells was significantly increased after DAC treatment (*P* value <0.05), but not by TSA treatment. The expression of STK3 increased only slightly in IOSE80 treated with DAC (*P* value <0.05) (Figure 3(b)). To validate the methylation-mediated downregulation of STK3 in ovarian cancer, we performed bisulfite sequencing PCR of DNA from IOSE80, CAOV3, OVCAR3, and OVCAR8 cells. The STK3 promoter in ovarian cancer cells showed significantly higher levels of methylation compared with that in IOSE80 (CAOV3: 43%; OVCAR3: 64%; and OVCAR8: 87%) (Figure 3(c)*P* value <0.05). These data indicated that epigenetic methylation suppresses the expression level of STK3 in ovarian cancer.

### 3.4. STK3 Correlates with CD8^+^ T-Cell in Ovarian Cancer

**Figure 4:**
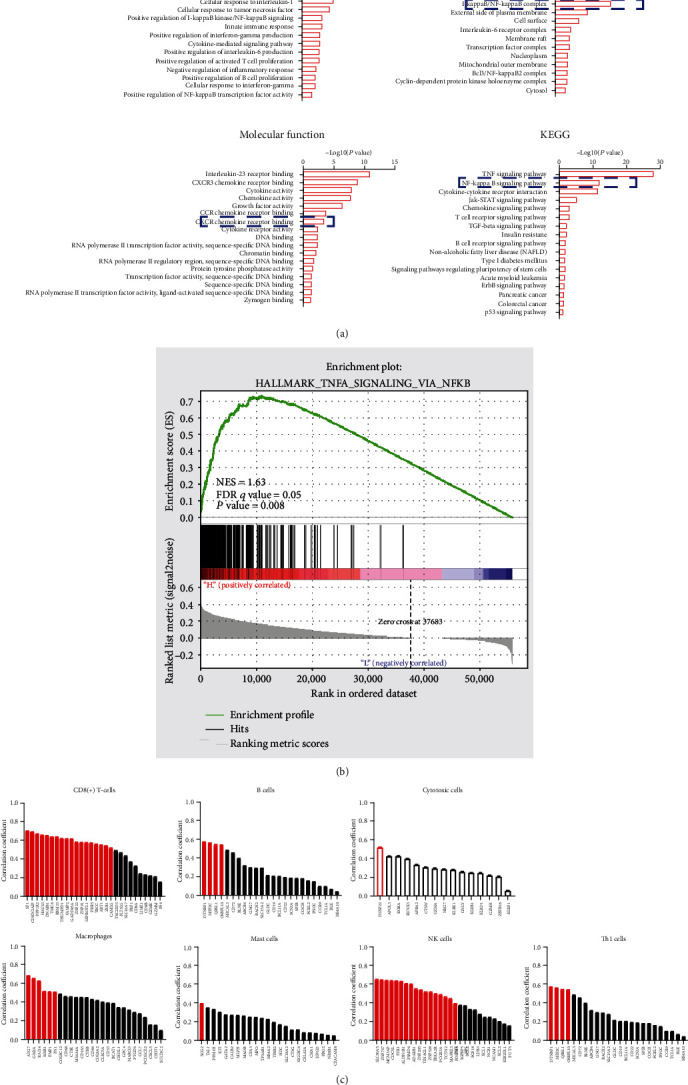
STK3 is correlated with CD8+ T cell infiltration and the NF-*κ*B signaling pathway in ovarian cancer. (a) Representative GO and KEGG categories affected by the STK3 expression in OVCAR8 cells. (b) Gene set enrichment analysis (GSEA) using reactome gene sets compared the TCGA ovarian database. NES: normalized enrichment score. (c) The correlation between STK3 and specific gene signatures of different immune cells.

To study the possible mechanism of STK3 in ovarian cancer, we compared the transcriptome of STK3 overexpressed ovarian cancer OVCAR8 cells with that of control cells by high-throughput transcriptome sequencing (RNA-seq, supplementary Table 1). The differentially expressed genes were analyzed by GO analysis and KEGG enrichment analysis. The results showed that overexpressing STK3 significantly affected multiple inflammatory responses, including I-kappaB kinase/NF-kappa B signaling, I-kappa B kinase/NF-kappa B signaling complex, CXCR chemokine receptor binding, and NF-kappa B signaling pathway, indicating a regulatory role of STK3 in ovarian cancer immune microenvironment (Figure 4(a)).

We used GSEA to analyze the differentiated genes in the high and low STK3 expression groups in the TCGA ovarian cancer database. The results indicated that the STK3 expression was related to the NF-*κ*B signaling pathway, which was TNFA_SIGNALING_VIA_NFKB. STK3 may regulate immune microenviroment of ovarian cancer cells by affecting NF-*κ*B signaling (Figure 4(b)).

To characterize the potential immune components in the tumor microenvironment affected by STK3, an immunome compendium was built by publicly available data from purified immune cell subsets. According to the previous studies, we investigated both innate immune cells (mast cells, macrophages, and natural killer cells) and adaptive immune cells (B cell, Th1, and CD8+ T cell) as well as cytotoxic cells[17]. We observed that STK3 was specifically correlated with the gene signatures of CD8+ T-cells. In contrast, no obvious correlation was found in the expression of STK3 and other immune component-related genes. STK3 might exhibit a regulatory role on CD8^+^ T cell infiltration in ovarian cancer (Figure 4(c)).

### 3.5. The Overexpression of STK3 Promoted the Release of CXCL16 and CX3CL1 and Migration of CD8+ T-Cells by Activating NF-*κ*B Signaling

**Figure 5:**
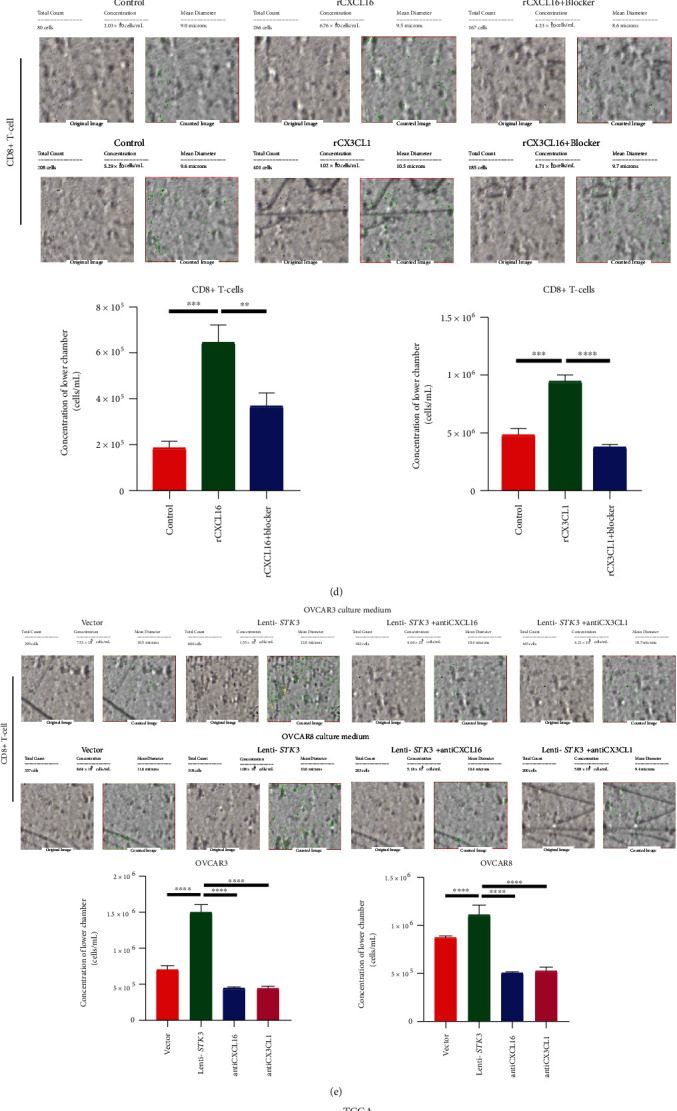
STK3 promoted release of CXCL16 and CX3CL1, migration of CD8+ T-cell, and activation of NF-*κ*B signaling. (a) Chemokine array of the conditional medium (CM) from vector and lenti-STK3 OVCAR8 cells. A. Table summarizing the relative signal intensity of indicated chemokines is presented in the lower left corner. (b) Real-time qPCR analysis of the effect of STK3 overexpression on the CXCL16 and CX3CL1 mRNA level in OVCAR3 and OVCAR8 cells (two-tailed Student's *t*-test, ^∗^*P* < 0.05; ^∗∗^*P* < 0.01). (c) ELISA analysis of the CXCL16 and CX3CL1 level in the CM from vector and lenti-STK3 OVCAR3 and OVCAR8 cells (two-tailed Student's *t*-test, ^∗∗^*P* < 0.01; ^∗∗∗^*P* < 0.001). (d) Effects of rCXCL16 and rCX3CL1 on the migratory ability of human CD8+ T-cells. The number of cells was counted by Cellometer (two-tailed Student's *t*-test, ^∗∗^*P* < 0.01; ^∗∗∗^*P* < 0.001; ^∗∗∗∗^*P* < 0.0001). (e) Ovarian cancer cell culture supernatants were neutralized with anti-CXCL16 antibody and anti-CX3CL1 antibody and the migratory ability of CD8+ T-cells. The number of cells was counted by Cellometer (two-tailed Student's *t*-test, ^∗∗^*P* < 0.01; ^∗∗∗^*P* < 0.001; ^∗∗∗∗^*P* < 0.0001). (f) Correlation between STK3 and NFKB1 in the TCGA database. (g) Correlation between NFKB1 and CXCL16 or CX3CL1 in the TCGA database. (h) Western blot analysis of the effect of the STK3 overexpression on the NF-*κ*B pathway activity.

To study the chemokines regulated by STK3 that promote CD8^+^ T-cell infiltration, the chemokine profiles of conditional medium from vector and lenti-STK3 OVCAR8 cells were analyzed using the RayBio Human Cytokine Antibody Array. Consistent with the results of RNA-seq (Supplementary Table 1), two cytokines, CXCL16 and CX3CL1, were significantly increased in the conditional medium of lenti-STK3 cells compared with vector cells (Figure 5(a)). Real-time PCR showed that the STK3 overexpression drastically promoted the mRNA level of CXCL16 and CX3CL1 in both OVCAR8 and OVCAR3 cells (*P* value <0.05) (Figure 5(b)). An ELISA assay confirmed the increase of CXCL16 and CX3CL1 in the conditional medium of lenti-STK3 cells (*P* value <0.05) (Figure 5(c)). We then evaluated whether these two chemokines are responsible for CD8^+^ T-cell infiltration in vitro. Stimulation of CD8^+^ T-cells with recombinant CXCL16 (rCXCL16) and CX3CL1 (rCXCL16) showed enhanced migratory ability, and this promotive effect was largely compromised by the addition of neutralizing anti-CXCL16 and anti-CX3CL1 antibody. This indicated that both CXCL16 and CX3CL1 contribute to CD8^+^ T-cells (*P* value <0.05) (Figure 5(d)). Consistently, neutralization of CXCL16 and CX3CL1 in the conditional medium from OVCAR8 and OVCAR3 cells also suppressed the migratory potential of CD8^+^ T-cells (*P* value <0.05) (Figure 5(e)).

To evaluate whether the NF-*κ*B pathway is responsible for the STK3-mediated CXCL16 and CX3CL1 expression, we first analyzed the correlation between the expression of STK3 and NF-*κ*B in TCGA. The levels of the STK3 expression were significantly positively correlated with NF-*κ*B (*P* value <0.05) (Figure 5(f)). Previous study revealed that NF-*κ*B is the widely reported transcription factor for CXCL16 and CX3CL1. Further analyses indicated that the level of NF-*κ*B was also significantly positively correlated with CXCL16 and CX3CL1 (*P* value <0.05) (Figure 5(g)). The phosphorylation level of p65 and NF-*κ*B inhibitor alpha (IkB*α*) in ovarian cancer regulated by STK3 was also investigated. The phosphorylation level of IkB*α* and p65 was markedly increased by the overexpression of STK3 (Figure 5(h)). These data indicated that the upregulated STK3 expression could promote the NF-*κ*B signaling of ovarian cancer, which may further induce the expression of CXCL16 and CX3CL1.

## 4. Discussion

Many studies have shown that key molecules of the Hippo signal transduction pathway are important in inhibiting the occurrence and development of malignant tumors [18]. Serine/threonine kinases (STKs) are key molecules in the Hippo pathway. STKs control organ growth and reduce tumor progression by their effects on the Hippo pathway [19].

In this study, bioinformatic analysis showed that STK3 has low levels in ovarian cancer tissues, and the reduced expression of STK3 is closely related to the poor prognosis of patients suffering ovarian cancer. As an important component of the Hippo pathway, STK3 has been involved in the progression of many types of cancers. For instance, the reduced expression of STK3 was found in gastric cancer, and tumors from patients with lymph node metastasis showed minimal levels of STK3 [20]. Genetic deletion of STK3 in hepatocytes also led to the development of hepatocellular carcinoma [21]. Additionally, STK3 inhibits the proliferation of breast cancer cells and is overexpressed in breast cancer tissues [22]. Increasing evidences showing that the STK3 alterations may be caused by genetic or epigenetic changes. For example, hypermethylation of the promoter region of the STK3 gene contributes to the downregulation of the STK3 expression in soft tissue sarcoma [23], which is consistent with our result on ovarian cancer. Therefore, we believe that STK3 may be a good prognostic marker for ovarian cancer.

It is known that the overexpression of STK3 can reduce the proliferation, migration, and invasion of glioblastoma and pancreatic cancer [24, 25]. In nonsmall cell lung cancer tissues, the overexpression of STK3 can reduce the invasion and metastasis ability of lung cancer cells and promote lung cancer cell apoptosis [26]. Similarly, in the present study, we found that the overexpression of STK3 inhibits the invasion, proliferation, and metastasis of ovarian cancer cells and promotes their apoptosis. Besides STK3, other components of the Hippo pathway have roles in ovarian cancer [27]. Previous study revealed that LATS1/2 downregulation by the ubiquitin system is associated with epithelial-mesenchymal transition-like phenotypic changes in ovarian cancer cells, which trigger abnormal migration and invasion [28]. Moreover, a relationship between high levels of nuclear YAP in primary tumor tissues and inferior survival in ovarian cancer patients has also been reported [29]. In immortalized ovarian surface epithelial cells, the overexpression of YAP increased cell proliferation and migration ability and promoted anchorage-independent growth as well as contributed to the resistance to cisplatin-induced apoptosis, suggesting the oncogenetic role of YAP in ovarian cancer. Our study further confirmed that STK3 may indirectly participate in suppressing ovarian cancer by regulating the activity of LATS1/2 and YAP.

CD8+ T-cells are considered to be antitumor immune cells [30]. We performed GO enrichment analysis and KEGG pathway enrichment analysis based on RNA-seq results of ovarian cancer cells in the control group and STK3 overexpression group. The results showed that STK3 and the NF-*κ*B signaling were related to infiltration of CD8+ T-cells. There is a close connection between the pathways. We also found a correlation between STK3 and CD8+ T-cells in the tumor microenvironment. The overexpression of STK3 can promote ovarian cancer cells to secrete CXCL16 and CX3CL1. The overexpression of STK3 can promote the migration of CD8+ T-cells. These results are the first correlation between the function of STK3 with CD8+ T-cells, and they help explain the effect of STK3 inhibition on the growth and metastasis of ovarian cancer from the perspective of the tumor microenvironment.

## 5. Conclusions

This study showed that upregulated serine/threonine kinase STK3 inhibits ovarian cancer aggressiveness and is correlated with CD8^+^ T-cells chemotaxis. These results provide insight into the roles of serine/threonine kinase in immune modulation. We also found that STK3 modulates intracellular NF-*κ*B signaling. These findings strongly suggest that the STK3/NF-*κ*B/CXCL16-CX3CL1/CD8^+^ T-cell axis is a potential therapeutic target in ovarian cancer.

## Figures and Tables

**Table 1:** The oligonucleotide sequence of primers used in this experiment.

Genes	Forward primer (5′-3′)	Reverse primer (5′-3′)
*STK3*	GCUGGAAAUAUUCUCCUUATT	UAAGGAGAAUAUUUCCAGCTT
*CXCL16*	CCCGCCATCGGTTCAGTTC	CCCCGAGTAAGCATGTCCAC
*CX3CL1*	ACTCTTGCCCACCCTCAGC	TGGAGACGGGAGGCACTC
*RPS18*	CAGCCAGGTCCTAGCCAATG	CCATCTATGGGCCCGAATCT

## Data Availability

The data used to support the findings of this study were supplied by Rong Zhang under license and so cannot be made freely available. Requests for access to these data should be made to Rong Zhang, rongzhang@163.com.

## Supplementary Materials

Supplementary Materials See Supplementary Table 1 in the Supplementary Material for comprehensive transcriptome analysis in control OVCAR8 cells and STK3-overexpressing OVCAR8 cells. Transcriptome analysis in vector control and STK3-overexpressing OVCAR8 cells. Transcriptome analysis was performed on vector control and STK3-overexpressing OVCAR8 cells after extracting total RNA. We included three biological repeats for each group in this experiment (*n* = 3). We performed paired-end sequencing using an Illumina NextSeq500 platform, and all samples were processed in the same sequencing run of the Illumina NextSeq 500 system and analyzed together with the aim to avoid the batches effect.
